# Solitary fibrous tumor of the nasal cavity

**DOI:** 10.1016/S1808-8694(15)30535-8

**Published:** 2015-10-18

**Authors:** Gisele Alborghetti Nai, Gabriel Cardoso Ramalho Neto

**Affiliations:** 1 Specialist, Professor; 2 Specialist, otorhinolaryngologist. Faculdade de Medicina - Universidade do Oeste Paulista (UNOESTE)

**Keywords:** nasal cavity, epistaxis, fibroid tumor

## INTRODUCTION

The solitary fibrous tumor (SFT) is a mesenchymal neoplasm first described by Klemperer and Rabin^1^ in 1931. It involves primarily the pleura, but has been described in the urinary bladder, limbs, thorax and abdomen, lungs, kidneys, prostate, meninges, mediastinum, and head & neck.^2^

In the head and neck, this tumor may involve the orbit, salivary glands, soft tissues, eyelids, mouth and nose, nasopharynx, retropharinx, oropharynx, and the thyroid gland.^3^,^4^

This study presents the fifth case of nasal SFT in the literature, the second in the Latin-American literature.

## CASE REPORT

**Figure 1:**
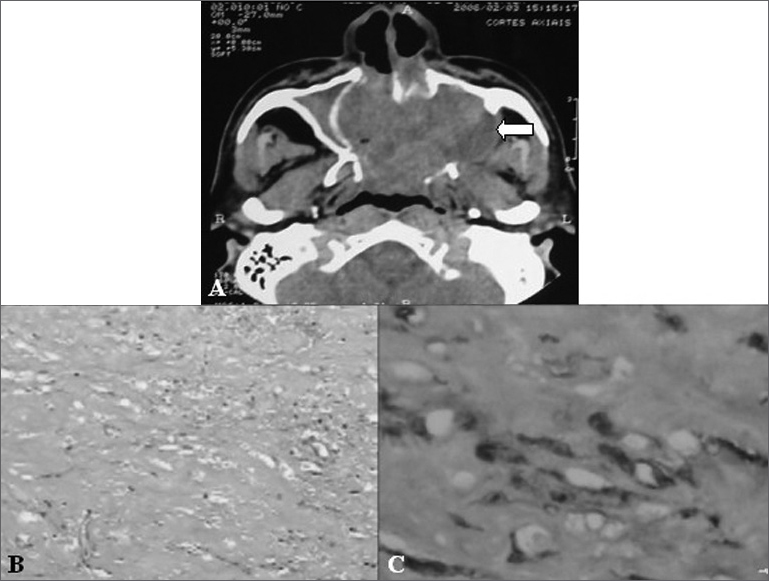
A - Cranial CT showing a large expansive tumor in the nasal fossa (arrow). B - Photomicroscopy showing a fusocellular and collagenic neoplasm with vascular areas (hematoxylin-eosin stained, 100x magnification). C - Immunohistochemistry showing positive neoplastic cells for the CD34 marker (400x magnification).

A male patient aged 54 years presented with epistaxis. Cranial computed tomography (CT) revealed a large expansive tumor in the nasal fossa (Fig. 1).

A biopsy was undertaken, but excessive bleeding during this procedure resulted in insufficient material for a diagnosis. Surgery was done for full removal of the tumor.

The macroscopic exam showed a well-defined, white and fibrous tumor measuring 6 × 4 × 4 cm; sections demonstrated a large hemorrhagic infarction.

Microscopy revealed a hypocellular neoplasm consisting of fusiform cells within a dense collagen stroma; there were many vascular areas (Fig. 2). No mitotic activity was evidenced in the tumor.

Immunohistochemistry defined the diagnosis of SFT by demonstrating positive neoplastic cells for CD34 and vimentin markers (Fig. 3), and negative results for AE1/AE3 cytokeratin (epithelial marker), HHF35 (smooth muscle actin) and the S100 protein (neural marker).

The patient progressed well postoperatively, with no recurrences one and a half year after surgery.

## DISCUSSION

SFT is an uncommon neoplasm. Many terms have been used to name it, reflecting the initial controversies around its histogenesis. This tumor was first considered as a submesothelial1 or mesothelial neoplasm; it is currently defined as a mesenchymal tumor with findings reflecting myopericytic, fibroblastic and myofibroblastic differentiation to justify extrapleural cases.

The diagnosis of this tumor may be difficult when it is not located in the pleura, given the variability of its histology. Typical microscopic findings are: storiform growth pattern, fusiform cells with no atypias, alternating dense cell and hypocellular areas and prominent branched vascularization similar to that of hemangiopericytomas. Histochemical studies diffusely express vimentin and CD34 protein, bcl-2 and CD99 focally, and are negative for muscle and epithelial cell markers.^2^

The present case is typical in term of incidence and tumor size in adults, with no gender preference, at a mean age of 50 years, measuring from 3 to 5 cm lengthwise.

Nasal SFTs usually result in nasal obstruction, occasionally epistaxis, rhinorrhea, anosmia, headache, facial pain, and visual disorders due to compression of the orbit.^3^ Our patient reported epistaxis, a symptom that had not been described in isolation.

Based on symptoms and radiological findings, the clinical differential diagnosis of nasal cavity STFs should be made with: fibrosarcoma, hemangiopericytoma, and nasopharyngeal carcinoma.

In the study case, due to tumor hypocellularity, extensive collagen areas, absence of cell atypias, and the patient's age, the differential diagnosis was made with: leiomyoma, myofibroma/myofibromatosis, fibroma and hemangiopericytoma. Leiomyomas are characterized by fusiform cells arranged in uniform anastomosing fascicles; immunohistochemically they express HHF35. Myofibromas/myofibromatosis and fibromas may be highly collagenic, and express vimentin, HHF35 and muscle-specific actin; they are CD34 negative. Hemangiopericytomas are more cellular and are focally and weakly positive for CD34 compared to STFs.

Full surgical removal is curative in most cases. The possibility of major bleeding during resection or in biopsies - as occurred in our case - should be taken into account.

The predominantly benign nature of nasal and extra-pleural STFs contrasts with the more aggressive behavior found in 23% of pleural tumors.^3^ The prognosis may be based on the presence or absence of histological findings of malignancy, such as significant cellularity, a mitotic index above 4 mitoses per 10 high magnification fields, the presence of necrosis and cellular pleomorphism. These findings, however, do not necessarily result in a clinically malignant behavior. A single nasal STF case described in the literature manifested malignancy, but was resected with no relapse.^4^ In the present case, histology suggested a benign course for the tumor, as in fact occurred.

## FINAL COMMENTS

STFs, although uncommon, should be remembered in the differential diagnosis of nasal cavity neoplasms; the definitive diagnosis is established by immunohistochemical tests and histopathology.
